# Ghrelin signaling in the cerebellar cortex enhances GABAergic transmission onto Purkinje cells

**DOI:** 10.1038/s41598-023-29226-3

**Published:** 2023-02-07

**Authors:** Moritoshi Hirono, Masanori Nakata

**Affiliations:** Department of Physiology, Wakayama Medical University, 811-1 Kimiidera, Wakayama, Wakayama 641-8509 Japan

**Keywords:** Neuroscience, Physiology, Endocrinology

## Abstract

Ghrelin, an orexigenic peptide ligand for growth hormone secretagogue receptor 1a (GHS-R1a), occurs not only in the stomach but also in the brain, and modulates neuronal activity and synaptic efficacy. Previous studies showed that GHS-R1a exists in the cerebellum, and ghrelin facilitates spontaneous firing of Purkinje cells (PCs). However, the effects of ghrelin on cerebellar GABAergic transmission have yet to be elucidated. We found that ghrelin enhanced GABAergic transmission between molecular layer interneurons (MLIs) and PCs using electrophysiological recordings in mouse cerebellar slices. This finding was consistent with the possibility that blocking synaptic transmission enhanced the ghrelin-induced facilitation of PC firing. Ghrelin profoundly increased the frequency of spontaneous inhibitory postsynaptic currents (IPSCs) in PCs without affecting miniature or stimulation-evoked IPSCs, whereas it significantly facilitated spontaneous firing of MLIs. This facilitation of MLI spiking disappeared during treatments with blockers of GHS-R1a, type 1 transient receptor potential canonical (TRPC1) channels and KCNQ channels. These results suggest that both activating TRPC1 channels and inhibiting KCNQ channels occur downstream the ghrelin-GHS-R1a signaling pathway probably in somatodendritic sites of MLIs. Thus, ghrelin can control PC firing directly and indirectly via its modulation of GABAergic transmission, thereby impacting activity in cerebellar circuitry.

## Introduction

Ghrelin, an endogenous orexigenic 28-amino acid acetylated peptide ligand, occurs in the stomach as a gastrointestinal hormone secreted into the circulation during fasting^1^, and transfers through the blood–brain barrier (BBB)^2^ and exist in the brain as well^3–5^. The peptide binds to the growth hormone secretagogue receptor type 1a (GHS-R1a) that is widely expressed in various brain areas^5–7^. Thus, ghrelin is now likely to play crucial roles in brain functions, such as the regulation of neuronal excitability and the modulation of synaptic transmission and plasticity^8^ in the central nervous system (CNS) that include neuropeptide Y neurons and growth hormone-releasing hormone neurons in the arcuate nucleus^3,9,10^, dopaminergic neurons in the substantia nigra pars compacta^11^, hippocampal pyramidal neurons^12^, and dorsal raphe nucleus neurons^13^. In the cerebellar cortex, both ghrelin and GHS-R1a are expressed^6,14,15^, and the activation of GHS-R1a on Purkinje cells (PCs), the sole outputs of the cerebellar cortex, increases the firing rate of spontaneous action potentials^16^. However, roles of ghrelin in cerebellar neurotransmission at excitatory and inhibitory synapses remain unknown.

GABAergic interneurons execute crucial synaptic functions via inhibitory transmission in the neuronal network^17^. GABAergic inhibitory transmission between molecular layer interneurons (MLIs) and PCs can regulate the patterns of simple spikes of PCs for cerebellar information processing and neural plasticity^18–20^ as well as control the excitatory/inhibitory balance in the cerebellar cortex^21^. Characterization of individual synaptic modulators in the cerebellum is crucial for better understanding of cerebellar motor learning and coordination^22^, however, the effects of ghrelin on inhibitory GABAergic transmission onto PCs have yet to be precisely elucidated. Thus, the aim of this study is to explore how ghrelin affects GABAergic transmission onto PCs and changes excitability of MLIs using electrophysiological recordings in mouse cerebellar slices.

We found that ghrelin enhanced spontaneous inhibitory postsynaptic currents (IPSCs) recorded from PCs, whereas the peptide did not alter miniature or evoked IPSCs in PCs. Moreover, ghrelin caused an increase in the firing rate of MLIs by activating type 1 transient receptor potential canonical (TRPC1) channels and inhibiting KCNQ channels. The results, therefore, suggest that GHS-R1a is expressed at somatodendritic sites of MLIs and its activation by ghrelin elicits the facilitation of firing in MLIs, whereas GHS-R1a signaling is unlikely to participate in controlling the release probability of GABA from axon terminals. Thus, ghrelin controls the firing level of PCs directly and indirectly via its modulation of GABAergic transmission within the cerebellar cortex.

## Results

### Effects of ghrelin on spontaneous firing of PCs

**Figure 1:**
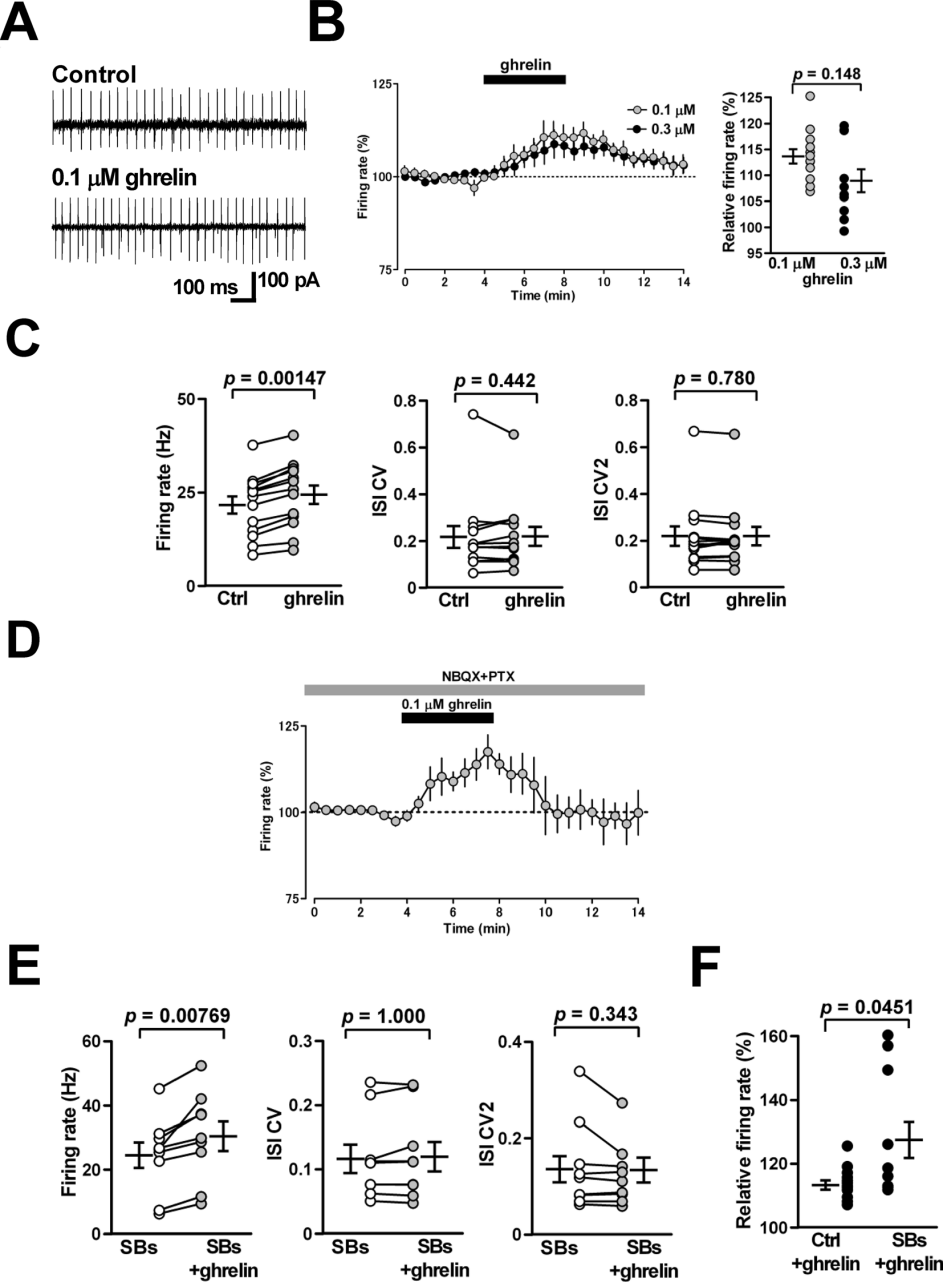
Effects of ghrelin on spontaneous firing of cerebellar PCs. (**A**) Representative traces of spontaneous firing recorded by the cell-attached mode from PCs before treatment (control), and in the presence of ghrelin (0.1 μM). (**B**) left: Time course of the firing rate of PCs with perfusion of ghrelin (0.1 μM: gray circles, 0.3 μM: black circles). The firing rate of PCs was expressed as a percentage of the baseline, which was determined for 4 min before application of ghrelin. Each point represents the mean values obtained from 13 cells for 0.1 μM and 12 cells for 0.3 μM. right: The magnitude of ghrelin-induced firing facilitation of PCs at 0.1 (n = 13, gray circles) and 0.3 μM (n = 12, black circles). (**C**) The peptide increased the firing rate of PCs significantly (left) without changing the coefficient of variation (CV) (middle) or CV2 (right) of the inter-spike interval (n = 13). (**D**) Effects of blockers for synaptic transmission on the ghrelin-induced firing facilitation of PCs. Time course of the firing rate of PCs in the presence of synaptic blockers (SBs), NBQX (5 μM) and PTX (100 μM). The firing rate of PCs was expressed as a percentage of the baseline, which was determined for 4 min before application of ghrelin. Each point represents the mean values obtained from 9 cells. (**E**) Ghrelin strongly increased the firing rate of PCs without changing the CV (middle) or CV2 (right) of the inter-spike interval (n = 9). (**F**) The magnitude of ghrelin-induced firing facilitation of PCs in the presence of blockers for synaptic transmission (SBs, n = 9) is larger than that in the absence of the blockers (Ctrl, n = 13).

A previous study has shown that ghrelin facilitates the spontaneous firing of PCs via the activation of GHS-R1a expressed in PCs of the rat cerebellum^16^. To confirm this result, we applied cell-attached recordings to mouse cerebellar PCs and observed their spontaneous firing with and without ghrelin. Perfusion of ghrelin (0.1 μM, the same concentration as reported previously^16^) significantly increased the firing rate from 21.7 ± 2.3 to 24.5 ± 2.5 Hz; 114 ± 1% of control; n = 13 from 13 slices/9 mice; *p* = 0.00147) (Fig. 1A–C). In this study, we examined spike train regularity of cerebellar neurons by measuring not only the coefficient of variation (CV) but also CV2, which detects variability less sensitive to changes in the mean firing rate^23^. The peptide (0.1 μM) did not alter the CV (from 0.218 ± 0.047 to 0.220 ± 0.040; n = 13 from 13/9; *p* = 0.442) or CV2 (from 0.221 ± 0.042 to 0.220 ± 0.040; n = 13 from 13/9; *p* = 0.780) of the inter-spike interval (Fig. 1C). The magnitude of its facilitation of PC firing turned into smaller at a higher concentration of 0.3 μM (from 19.4 ± 2.0 to 21.2 ± 2.3 Hz; 109 ± 2% of control; n = 12 from 12/6; *p* = 0.00287), but there was no significant difference (*p* = 0.148) (Fig. 1B right). When we performed this recording at more physiological temperature (30–31 ˚C), 0.1 μM ghrelin similarly increased the firing rate of PCs from 45.9 ± 4.0 to 50.9 ± 4.0 Hz (111 ± 2% of control; n = 7 from 7/3; *p* = 0.00178).

We compared the extent of ghrelin-induced increase in the firing rate of PCs in the presence or absence of blockers of synaptic transmission. In the presence of synaptic blockers, the AMPA receptor antagonist 2,3-dioxo-6-nitro-1,2,3,4-tetrahydrobenzo[*f*]quinoxaline-7-sulfonamide disodium salt (NBQX) (5 μM) and the GABA_A_ receptor antagonist picrotoxin (PTX, 100 μM), ghrelin still caused an increase in the firing rate of PCs (from 24.6 ± 4.0 to 30.5 ± 4.6 Hz; 129 ± 7%; n = 9 from 9/4; *p* = 0.00769) (Fig. 1D and E), suggesting that the ghrelin-induced firing facilitation of PCs did not require fast synaptic transmission, as reported in growth hormone-releasing hormone neurons in the arcuate nucleus^10^. Ghrelin did not change either the CV (from 0.117 ± 0.022 to 0.120 ± 0.023; n = 9 from 9/4; *p* = 1.000) or CV2 (from 0.136 ± 0.027 to 0.134 ± 0.026; n = 9 from 9/4; *p* = 0.343) of the inter-spike interval (Fig. 1E). The magnitude of increase was significantly higher with than without the synaptic blockers (129 ± 7% in the synaptic blockers-containing artificial cerebrospinal fluid [ACSF] vs. 114 ± 1% in the normal ACSF; *p* = 0.0451) (Fig. 1F). This result supports a possibility that ghrelin suppresses excitatory synaptic transmission and/or enhances GABAergic transmission onto PCs.

### Ghrelin has no effect on PF-EPSCs or evoked IPSCs in PCs

**Figure 2:**
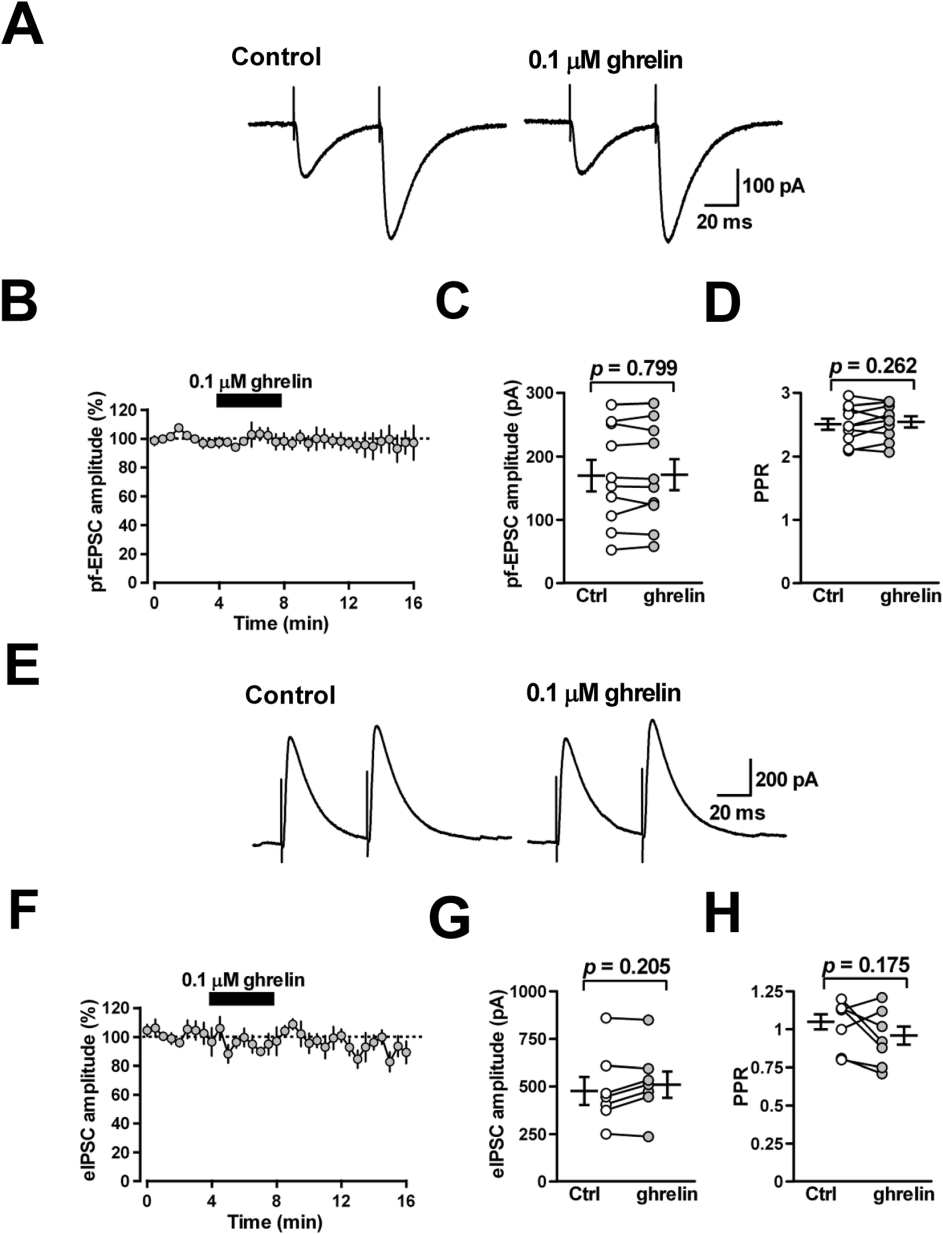
Effects of ghrelin on stimulation-evoked PF-EPSCs and IPSCs in PCs. (**A**) Representative averaged traces of four consecutive PF-EPSCs recorded from PCs held at –60 mV before treatment (control), and in the presence of ghrelin (0.1 μM). (**B**) Time course of the peak amplitude of the first PF-EPSCs recorded from PCs. The first PF-EPSC amplitude was expressed as a percentage of the baseline, which was determined for 4 min before application of ghrelin. Each point represents the mean values obtained from 10 cells. (**C**, **D**) The peptide affected neither the amplitude (**C**) nor the paired-pulse ratio (PPR) (**D**) of PF-EPSCs (*n* = 10). (**E**) Representative averaged traces of four consecutive eIPSCs recorded from PCs held at 10 mV before treatment (control), and in the presence of ghrelin (0.1 μM). (**F**) Time course of the peak amplitude of the first eIPSCs recorded from PCs. The first eIPSC amplitude was expressed as a percentage of the baseline, which was determined for 4 min before application of ghrelin. Each point represents the mean values obtained from 7 cells. (**G**) (**H**) The peptide affected neither the amplitude (**G**) nor the paired-pulse ratio (PPR) (***H***) of eIPSCs (*n* = 7).

To examine the effects of ghrelin on stimulation-evoked synaptic transmission onto PCs in the cerebellar cortex, we first recorded excitatory postsynaptic currents (EPSCs) in PCs induced at parallel fiber (PF)-PC synapses. PCs were held at − 60 mV and PF-EPSCs were induced by focal stimulation applied to the ML. Ghrelin (0.1 μM), applied by superfusion for 4 min, did not alter the amplitude (from 170.1 ± 24.9 to 171.3 ± 24.7 pA; 102 ± 3% of control; *n* = 10 from 10/6; *p* = 0.799) or the paired-pulse ratio (PPR) of PF-EPSCs (from 2.51 ± 0.09 to 2.55 ± 0.09; 102 ± 2% of control; *n* = 10 from 10/6; *p* = 0.262) (Fig. 2A–D), indicating that ghrelin does not change stimulation-evoked excitatory synaptic transmission at PF-PC synapses.

We then investigated whether ghrelin affected eIPSCs in PCs. Recordings were obtained from PCs held at 10 mV, and eIPSCs were induced by focal stimulation applied to the ML. Bath-application of ghrelin (0.1 μM) altered neither the amplitude (from 477.0 ± 74.2 to 510.4 ± 69.5 pA; 108 ± 4% of control; *n* = 7 from 7/5; *p* = 0.205) nor the paired-pulse ratio (from 1.03 ± 0.06 to 0.94 ± 0.07; 92 ± 5% of control; *n* = 7 from 7/5; *p* = 0.175) of eIPSCs (Fig. 2E–H), indicating that ghrelin does not change stimulation-evoked IPSCs in PCs. These results suggest that short-term application of ghrelin does not change efficacy of transmission at either excitatory PF-PC or inhibitory MLI-PC synapses.

### Ghrelin facilitates spontaneous IPSCs in PCs

**Figure 3:**
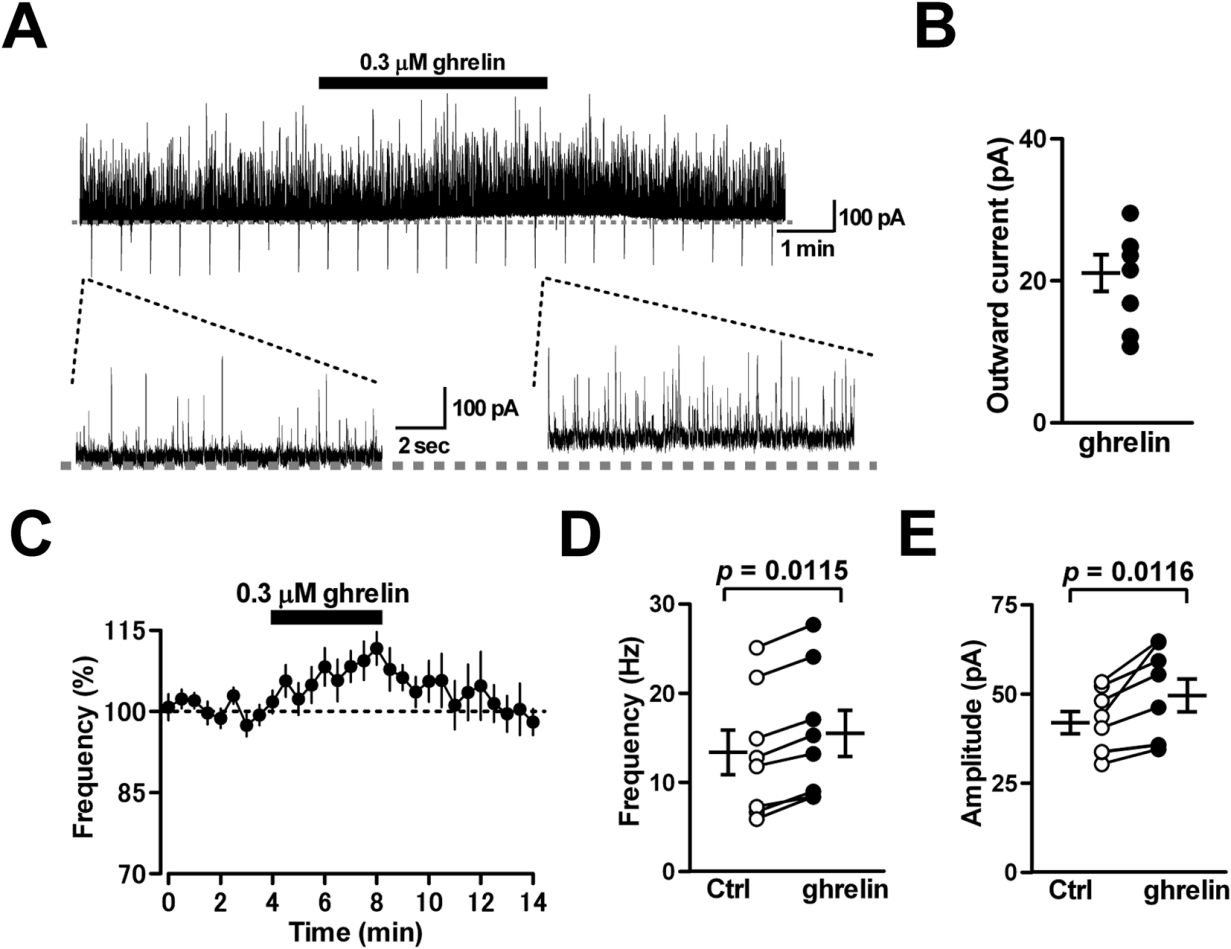
Effects of ghrelin on spontaneous IPSCs in PCs. (**A**) Representative traces of sIPSCs recorded from PCs held at − 35 mV before treatment, and in the presence of ghrelin (0.3 μM). Gray dotted lines indicate the basal current level before application of the peptide. The lower traces represent sIPSCs displayed on an expanded time scale before and during the application of ghrelin. (**B**) Ghrelin caused an outward shift of baseline currents (n = 8). (**C**) Time course of the frequency of sIPSCs in PCs. The sIPSC frequency was expressed as a percentage of the baseline, which was determined for 4 min before application of ghrelin. Each point represents the mean values obtained from 8 cells. (**D**, **E**) The peptide increased both the frequency (**D**) and the amplitude (**E**) of sIPSCs significantly (n = 8).

We sought to examine whether ghrelin modulates sIPSCs in PCs, because tonic GABAergic synaptic inhibition regulates firing patterns of PCs^18^. We recorded sIPSCs from PCs held at –35 mV using whole-cell recordings with a K^+^-based internal solution. Here, we tested the effects of 0.3 μM ghrelin on the currents, because it is conceivable that at this concentration ghrelin likely suppresses the ghrelin-induced firing facilitation of PCs clearly as shown in Fig. 1B. Application of ghrelin (0.3 μM) for 4 min increased both the frequency (from 13.4 ± 2.5 to 15.5 ± 2.6 Hz; 120 ± 4% of control; *n* = 8 from 8/4; *p* = 0.0115) and the amplitude (from 42.0 ± 3.1 to 49.6 ± 4.6 pA; 117 ± 5% of control; *n* = 8 from 8/4; *p* = 0.0116) of sIPSCs (Fig. 3A and C–E). Because the sIPSC frequency is increased, and considering the width of a single sIPSC event, the increase in the sIPSC amplitude could be attributed to the increment of the probability of overlapping single sIPSCs. Perfusion of ghrelin also caused a slow outward response of the baseline current (21.1 ± 2.6 pA; *n* = 8 from 8/4) (Fig. 3A and B). CGP55845 (2 μM), a GABA_B_ receptor antagonist, did not block the outward current (17.5 ± 1.8 pA; *n* = 5 from 5/2), implying that ghrelin induces slow hyperpolarization of PCs directly via open G-protein-coupled inward-rectifying K^+^ (GIRK) channels and/or non-GIRK channels^24,25^. These results indicate that, in addition to its excitatory action on PCs, ghrelin facilitates spontaneous GABAergic transmission onto PCs and causes slow inhibitory responses in PCs, resulting in the attenuation of the firing facilitation of PCs. The mechanism underlying this sIPSC facilitation could be ghrelin-induced excitation of MLIs because the peptide did not alter eIPSCs in PCs, as shown in Fig. 2E–H.

### Miniature IPSCs in PCs are insensitive to ghrelin

**Figure 4:**
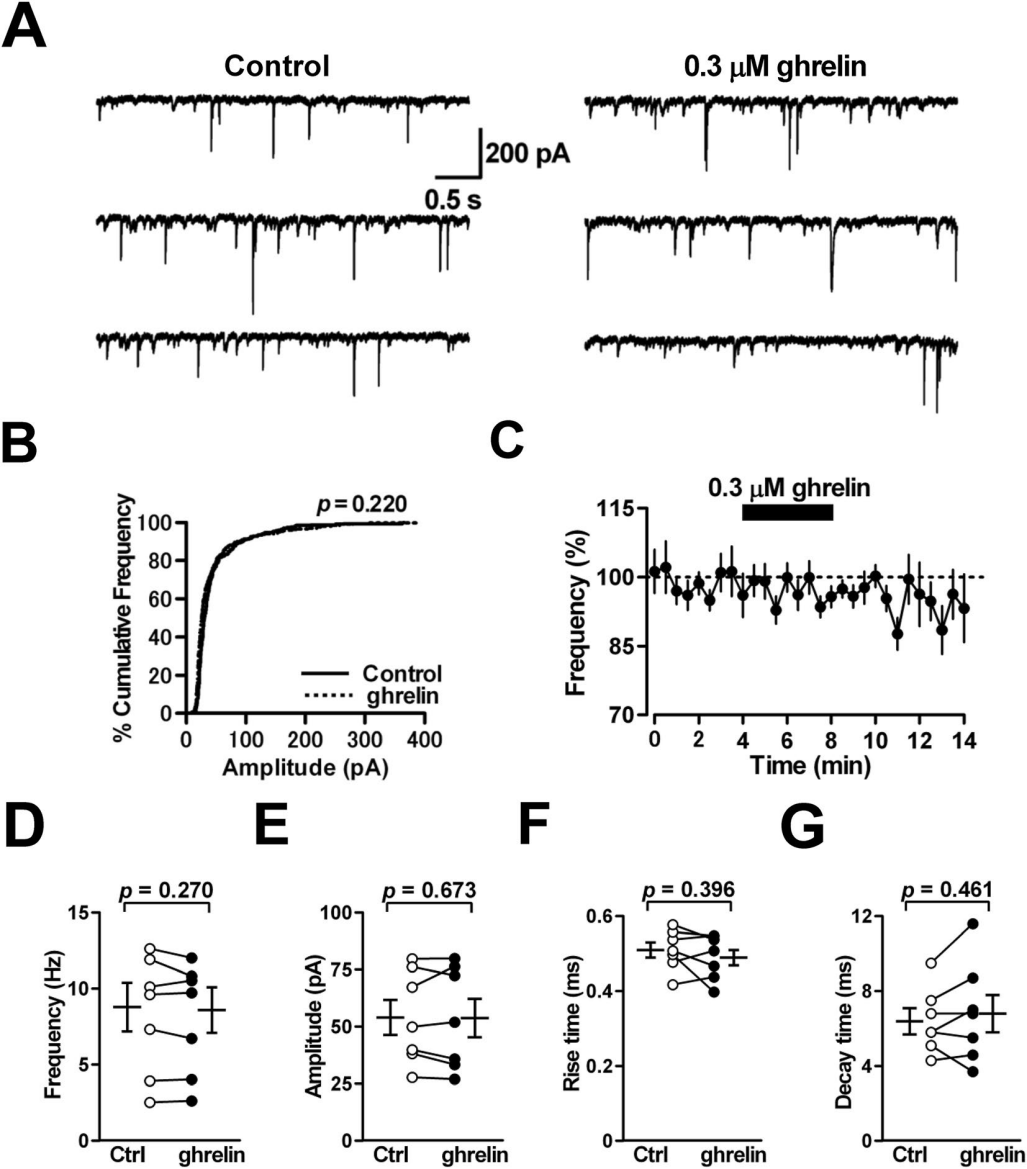
Effects of ghrelin on miniature IPSCs in PCs. (**A**) Representative traces of mIPSCs recorded from a PC before treatment (control) and during the application of ghrelin (0.3 μM). (**B**) Cumulative probability fractions of the mIPSC amplitude were obtained from the same cell as in (**A**). Amplitude distribution was not affected by ghrelin (*P* = 0.220 by Kolmogorov–Smirnov test). (**C**) The time course of the mIPSC frequency in PCs. The mIPSC frequency was expressed as a percentage of the baseline, which was determined for 4 min before application of ghrelin. Each point represents the mean values obtained from seven cells. (**D**–**G**) Ghrelin had no effect on the frequency (**D**), amplitude (**E**), rise time (**F**) or decay time (**G**) of mIPSCs in PCs (*n* = 7).

As shown above, ghrelin is devoid of any actions on GABAergic transmission at MLI-PC synapses, which hints that the peptide did not impact the release probability of GABA from axon terminals of MLIs, the number and the sensitivity of GABA_A_ receptors at postsynaptic PCs. To confirm this possibility, we examined whether 0.3 μM ghrelin influences mIPSCs in PCs held at –60 mV. Bath-application of the peptide altered neither the frequency (from 8.8 ± 1.6 to 8.6 ± 1.5 Hz; 98 ± 2% of control; *n* = 7 from 7/4; *p* = 0.270) (Fig. 4A, C and D) nor the amplitude of mIPSCs (from 54.1 ± 7.7 to 53.8 ± 8.5 pA; 98 ± 3% of control; *n* = 7 from 7/4; *p* = 0.673) (Fig. 4A and E) (*p* = 0.220, by Kolmogorov–Smirnov test; Fig. 4B). Application of ghrelin did not alter the rise time (from 0.51 ± 0.02 to 0.49 ± 0.02 ms; 97 ± 3% of control; *n* = 7 from 7/4; *p* = 0.396) or the decay time of mIPSCs (from 6.4 ± 0.7 to 6.8 ± 1.0 ms; 105 ± 7% of control; *n* = 7 from 7/4; *p* = 0.461) (Fig. 4F and G). These results suggest that ghrelin cannot impact both the release probability of GABA from axon terminals of MLIs and the sensitivity of GABA_A_ receptors at postsynaptic PCs, presumably because the ghrelin receptor GHS-R1a is not expressed at either axon terminals of MLIs or the inhibitory postsynaptic sites of PCs, but is only expressed at somatodendritic sites of MLIs.

### Ghrelin facilitates spontaneous firing of MLIs

**Figure 5:**
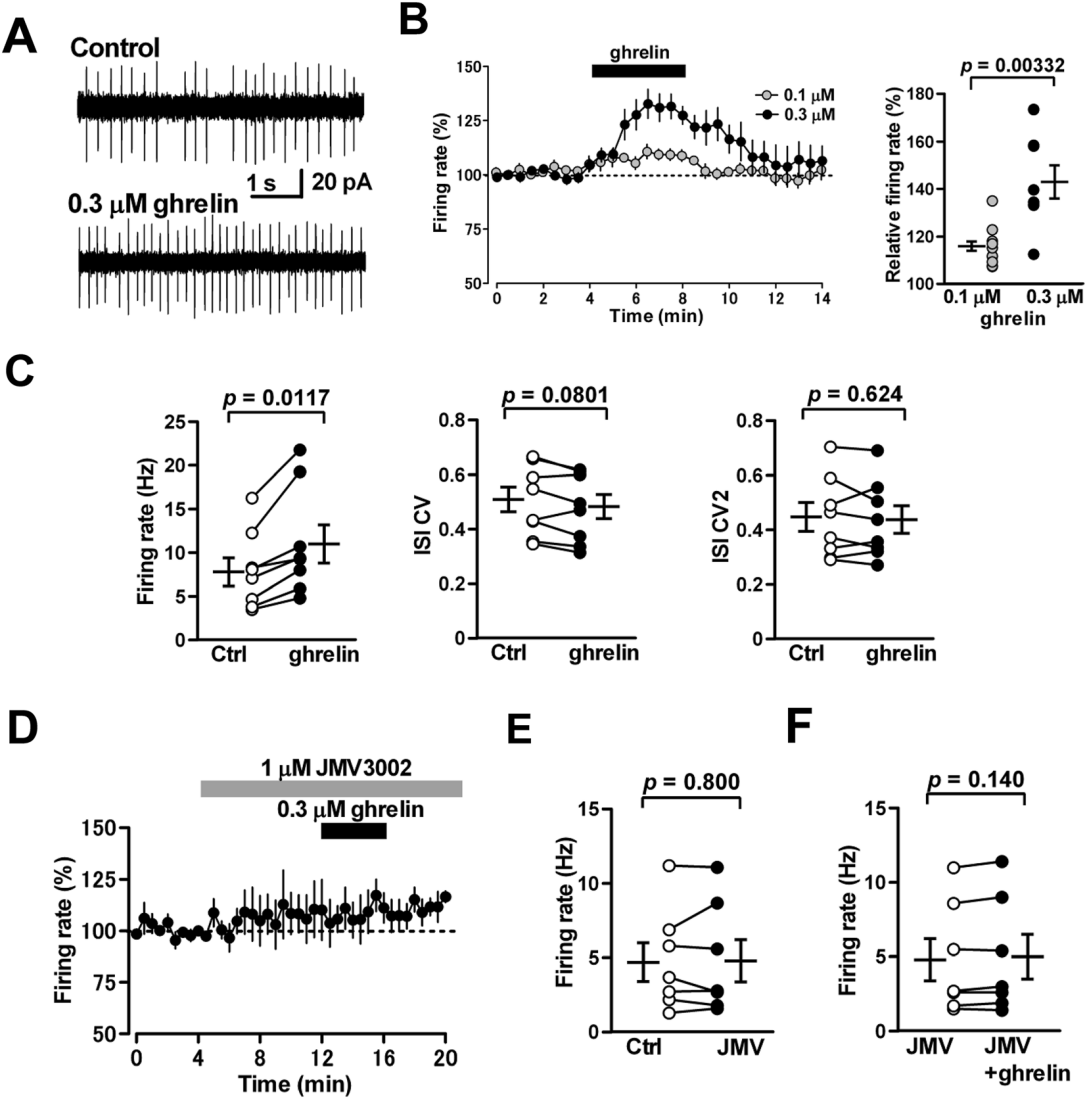
Effects of ghrelin on spontaneous firing of MLIs. (**A**) Representative traces of spontaneous action potentials of MLIs recorded by a cell-attached recording before treatment (control) and during the application of ghrelin (0.3 μM). (**B**) left: Time course of the firing rate of MLIs with perfusion of ghrelin (gray circles: 0.1 μM, black circles: 0.3 μM). The firing rate was expressed as a percentage of the baseline, which was determined for 4 min before application of ghrelin. Each point represents the mean values obtained from 12 cells for 0.1 μM and 8 cells for 0.3 μM. right: The magnitude of ghrelin-induced firing facilitation of MLIs at 0.1 (n = 12, gray circles) and 0.3 μM (n = 8, black circles). (**C**) Ghrelin increased the firing rate of MLIs (left) without changing the CV (middle) or CV2 (right) of the inter-spike interval (n = 8). (**D**) Time course of the firing rate of MLIs. The GHS-R1a antagonist JMV3002 (1 μM) was applied during the periods indicated by a horizontal gray bar. The firing rate of MLIs was expressed as a percentage of the baseline, which was determined for 4 min before application of the antagonist. Each point represents the mean values obtained from 7 cells. (**E**, **F**) The GHS-R1a antagonist did not affect the baseline firing rate (**E**), while it inhibited the ghrelin-induced firing facilitation of MLIs (***F***) (n = 7).

To further clarify whether the ghrelin-mediated potentiation of sIPSCs is attributed to ghrelin-induced firing facilitation of MLIs, we applied cell-attached recordings to MLIs and recorded the firing rate of MLIs. Ghrelin (0.3 μM), when applied for 4 min by perfusion, increased the firing rate of MLIs (from 7.8 ± 1.6 to 11.0 ± 2.2 Hz; 143 ± 7% of control; *n* = 8 from 8/6; *p* = 0.0117) (Fig. 5A–C). Ghrelin did not change either the CV (from 0.510 ± 0.046 to 0.484 ± 0.044; n = 8 from 8/6; *p* = 0.0801) or CV2 (from 0.448 ± 0.053 to 0.439 ± 0.050; n = 8 from 8/6; *p* = 0.624) of the inter-spike interval (Fig. 5C). The magnitude of its facilitation of MLI firing turned into significantly smaller at a lower concentration of 0.1 μM (from 4.5 ± 0.7 to 5.1 ± 0.7 Hz; 116 ± 2% of control; n = 12 from 12/5; p = 0.00209) than that at 0.3 μM (116 ± 2% of control vs 143 ± 7% of control;* p* = 0.00332) (Fig. 5B right). When we performed this recording at more physiological temperature (30–31 ˚C), 0.3 μM ghrelin similarly facilitated MLI firing from 6.4 ± 2.3 to 7.5 ± 2.5 Hz (124 ± 6% of control; n = 5 from 5/2; *p* = 0.0431).

Pretreatment of MLIs with the antagonist for GHS-R1a JMV3002 (1 μM) did not change the baseline firing rate of MLIs (from 4.7 ± 1.3 to 4.8 ± 1.4 Hz; 100 ± 8%; n = 7 from 7/4; *P* = 0.800) (Fig. 5D and E), suggesting that endogenous ghrelin has no effect on MLI firing. The antagonist inhibited the ghrelin-induced firing facilitation of MLIs (from 4.8 ± 1.4 to 5.0 ± 1.5 Hz; 103 ± 3%; n = 7 from 7/4; *p* = 0.140) (Fig. 5D and F), suggesting that ghrelin activates GHS-R1a on MLIs, resulting in the increase in their firing rates.

**Figure 6:**
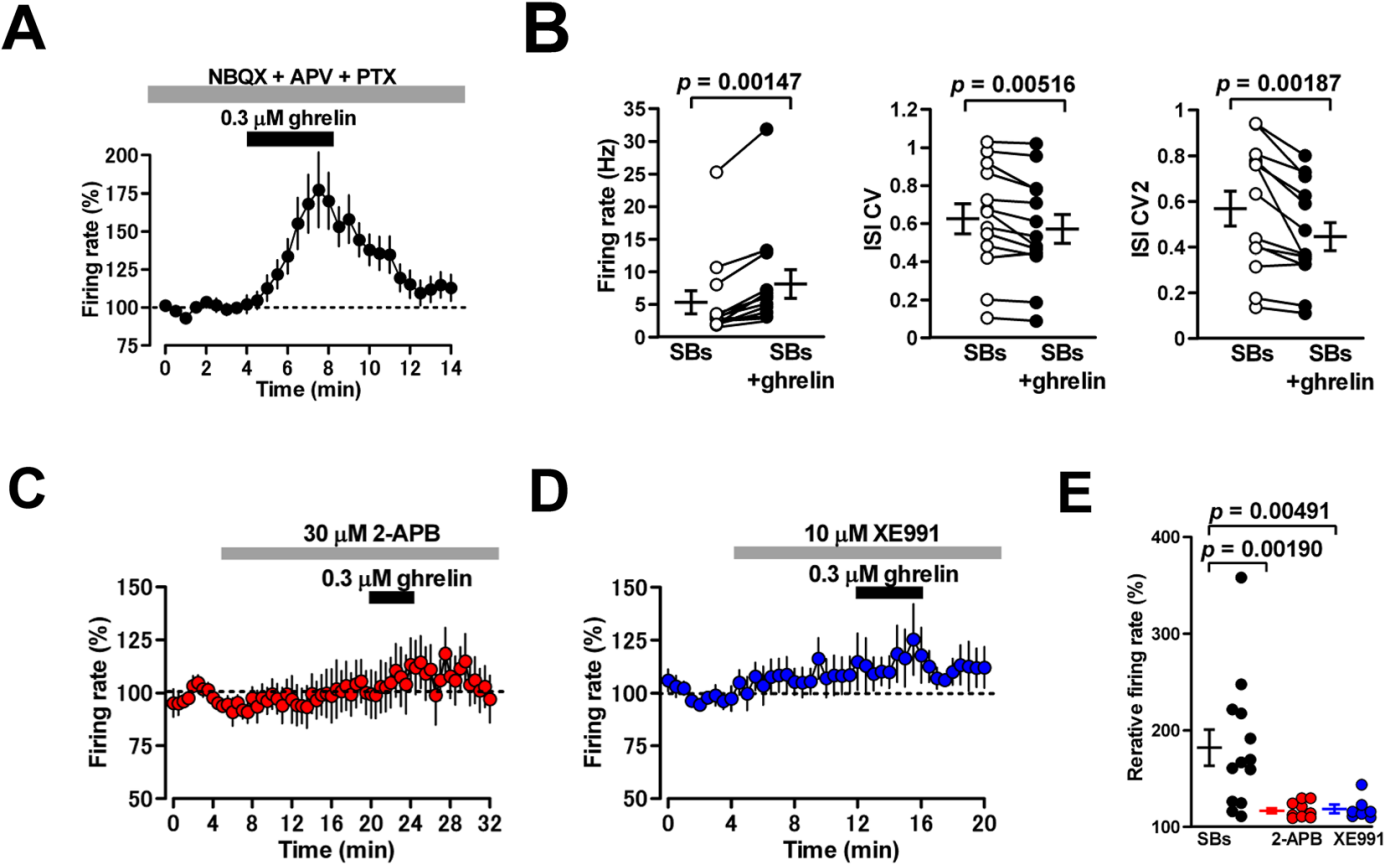
Effects of inhibitors for channels possibly involved in ghrelin signaling downstream on ghrelin-induced firing facilitation of MLIs. (**A**) Time course of the firing rate of MLIs in the presence of blockers for synaptic transmission (SBs), NBQX (5 μM), APV (15 μM), and PTX (100 μM). The firing rate of MLIs was expressed as a percentage of the baseline, which was determined for 4 min before application of ghrelin. Each point represents the mean values obtained from 13 cells. (**B**) Ghrelin strongly increased the firing rate of MLIs (left) changing significantly both the CV (middle) and CV2 (right) of the inter-spike interval (n = 13). (**C**) Effects of a non-selective TRPC channel inhibitor 2-APB (30 μM) on the ghrelin-induced firing facilitation of MLIs. Time course of the firing rate of MLIs. Each point represents the mean values obtained from 9 cells. (**D**) Effects of a blocker for KCNQ channels XE991 (10 μM) on the ghrelin-induced firing facilitation of MLIs. Time course of the firing rate of MLIs. Each point represents the mean values obtained from 7 cells. (**E**) The magnitudes of ghrelin-induced firing facilitation of MLIs in the presence of 2-APB and XE991 were lower than that in the absence of blocker.

Various signaling pathways have been reported to be involved in downstream of GHS-R1a activation. The activation of GHS-R1a facilitates G_q/11_ and β-arrestin cascades^26–29^, and our previous study reported that the activation of both can consequently open TRPC1 channels in MLIs and increase their firing rate^30^. To examine whether the ghrelin-induced firing facilitation of MLIs is attributed to the activation of TRPC1 channels, we applied a non-selective TRPC channel blocker 2-aminnoethoxydiphenylborane (2-APB) (30 μM) to cerebellar slices. Here, to evaluate the effects of ghrelin on intrinsic firing of MLIs, we bath-applied blockers of synaptic transmission, NBQX (5 μM), DL-2-amino-5-phosphonovaleric acid (APV) (15 μM), and PTX (100 μM). In the presence of these synaptic blockers, ghrelin increased the firing rate of MLIs (from 5.4 ± 1.8 to 8.2 ± 2.2 Hz; 182 ± 19%; n = 13 from 13/7; *p* = 0.00147) (Fig. 6A and B). The percentage of increase appeared to be enhanced compared to those obtained without these blockers, but not significantly (182 ± 19% with the synaptic blockers vs 143 ± 7% without the synaptic blocker; *p* = 0.180). During blocking synaptic transmission, ghrelin changed spike train regularity of MLI firing (CV: from 0.627 ± 0.079 to 0.573 ± 0.076; n = 13 from 13/7; *p* = 0.00516, CV2: from 0.570 ± 0.077 to 0.447 ± 0.061; n = 13 from 13/7; *p* = 0.00187) (Fig. 6B). 2-APB slightly decreased the baseline firing rate of MLIs (from 8.3 ± 1.2 to 7.7 ± 1.3 Hz; 92 ± 4%; n = 9 from 9/5; *p* = 0.353). Although the peptide increased the firing rate (from 7.7 ± 1.3 to 8.9 ± 1.4 Hz; 117 ± 3% of control; n = 9 from 9/4; *P* = 0.00769) (Fig. 6C), 2-APB reduced the magnitude of ghrelin-induced firing facilitation (117 ± 3% of the control vs 182 ± 19% of the control; *p* = 0.00190) (Fig. 6E). Therefore, the activation of TRPC channels consisting of TRPC1 isoforms contributes to the ghrelin-induced firing facilitation of MLIs. Furthermore, an alternative mechanism underlying the ghrelin-induced firing facilitation is the suppression of Kv7/KCNQ channels via G_q/11_^12^. It has been reported that KCNQ2 and KCNQ3 are expressed in axon initial segments of MLIs^31^. When we applied the KCNQ blocker 10,10-*bis*(4-pyridinylmethyl)-9(10*H*)-anthracenone (XE991)** (**10 μM) to cerebellar slices, it slightly increased the baseline firing rate of MLIs (from 4.7 ± 1.0 to 4.8 ± 0.8 Hz; 108 ± 8%; n = 7 from 7/3; *P* = 0.289). Ghrelin still facilitated firing of MLIs (4.8 ± 0.8 to 5.6 ± 0.8 Hz; 119 ± 4% of control; n = 7 from 7/3; *p* = 0.0178) (Fig. 6D), but the blocker suppressed the magnitude of ghrelin-induced firing facilitation of MLIs (119 ± 4% of the control vs 182 ± 19% of the control; *p* = 0.00491) (Fig. 6E). Thus, the ghrelin-mediated inhibition of KCNQ channels is also involved in the firing facilitation of MLIs.

## Discussion

Our study shows that ghrelin facilitates inhibitory GABAergic transmission at MLI-PC synapses by an increase in the frequency of spontaneous action potentials of MLIs, while the peptide does not alter excitatory synaptic transmission at PF-PC synapses. Ghrelin potentiated sIPSCs in PCs but altered neither miniature nor evoked IPSCs, suggesting that ghrelin increases the excitability of MLIs without changing efficacy of GABAergic transmission at sites restricted to synapses between MLIs and PCs. Thus, it is most likely that GHS-R1a is expressed exclusively at somatodendritic sites of MLIs. This is the first study to characterize the effects of GHS-R1a activation on cerebellar GABAergic transmission.

GHS-R1a on MLIs could be activated by plasma ghrelin, which is elevated upon fasting^1^. The constitutive activation of GHS-R1a or its chronic activation by plasma ghrelin may cause continuous excitation of MLIs^32^. Our data that administration of the GHS-R1a antagonist JMV3002 did not change the baseline firing rate of MLIs may exclude the later possibility, or alternatively, this result may be simply due to the cerebellar slice preparations. Since ghrelin-immunopositive cells have been observed in the cerebellum of African ostriches^14^, it is conceivable that endogenous ghrelin synthesized and released from cerebellar neurons could stimulate GHS-R1a on MLIs. To address this possibility, further studies are necessary. Ghrelin modifies brain functions by altering intrinsic neuronal excitability and synaptic transmission in other brain areas. Although this peptide primarily enhances the membrane excitability of neurons^3,9,10,13,16^, it is also shown to reduce the neuronal excitability of CA1 pyramidal neurons in the dorsal hippocampus^33^. Additionally, ghrelin signaling controls synaptic plasticity of both excitatory and inhibitory transmissions^8^. At excitatory synaptic sites, the peptide causes an increase in spine density during long-term potentiation (LTP)^34^, enhancement of LTP in rat hippocampal CA1^35^, and modulation of AMPA receptor trafficking through ligand-independent activity^36,37^. On the other hand, at inhibitory synaptic sites, ghrelin has both facilitative and suppressive effects on GABAergic transmission^32,38,39^. Here we did not observe any alterations in the efficacy of transmission at either excitatory PF-PC or inhibitory MLI-PC synapses by short-term application of ghrelin. One possible reason for this is that GHS-R1a is not expressed locally at either pre- or post-synaptic sites of them. Another possible reason for synaptic transmission at PF-PC postsynaptic sites is that ghrelin activates signaling pathways simultaneously, which have opposite effects on the efficacy of the excitatory transmission through the activation of G_i/o_ and G_q/11_^27–29^, and consequently, counteract alterations in the efficacy. GHS-R1a activation commonly stimulates G_q/11_^40^, whose downstream signaling is required for the induction of long-term depression (LTD) at PF-PC synapses^22^. Furthermore, intracellular Ca^2^^+^ elevation, likely caused by ghrelin-mediated facilitation of PC firing^41^, may also support LTD induction. On the other hand, GHS-R1a activation also activates βγ subunits of G_o_-proteins, resulting in PKA-mediated suppression of Ca_v_2.1 voltage-gated Ca^2+^ channels^16^. This signaling pathway possibly reduces intracellular Ca^2+^ and contributes to the induction of LTP^42,43^. Additionally, a previous study reported that inhibition from MLIs promotes LTP at PF-PC synapses^44^; thus, the ghrelin-mediated facilitation of sIPSCs could contribute to LTP-induction. Therefore, ghrelin signaling in the cerebellar cortex can modulate the balance between LTD and LTP. Although in the present study we did not bath-apply ghrelin for longer time (e.g., several hours) to mimic hunger conditions, it is likely that such ghrelin treatment by itself elicits LTD or LTP at PF-PC synapses. Alternatively, ghrelin may only support LTD- or LTP-induction through the consequences of activation of the aforementioned signaling.

What are the molecular mechanisms underlying the effects of ghrelin on cerebellar MLIs? Because GHS-R1a can couple with several types of G proteins^27–29^, many intracellular signaling mechanisms can be raised for the ghrelin-mediated neuronal excitation. One of them is the blockade of Ca_v_2.1 and Ca_v_2.2^38,45^, which is also observed in cerebellar PCs^16^. Although axon terminals of MLIs are known to possess these types of Ca^2+^ channels, whose activation triggers GABA release^46,47^, ghrelin did not alter evoked or miniature IPSCs in PCs, indicating that GHS-R1a is not expressed at the axon terminals of MLIs or that ghrelin signaling does not function there. An alternative mechanism is the inhibition of Kv7/KCNQ currents via the GHS-R1a-PLC-PKC pathway, as has been shown in dopaminergic neurons of the rat substantia nigra^12^. Since axon initial segments of MLIs contain KCNQ2 and KCNQ3^31^, we tested the KCNQ blocker XE991 and observed that it attenuated ghrelin-induced firing facilitation of MLIs. Furthermore, MLIs also express TRPC1 channels, which open and facilitate firing through the downstream signaling of Gαq and β-arrestin^30^, both of which are activated by GHS-R1a stimulation. Administration of the non-selective TRP channel inhibitor 2-APB suppressed the ghrelin-induced firing facilitation of MLIs. Taken together, the ghrelin-induced firing facilitation of MLIs is attributed to not only the inhibition of KCNQ channels but also the activation of TRPC1 channels. Because bath-application of XE991 increased MLI firing slightly, it possibly occludes the ghrelin-induced firing facilitation depending on the activation of TRPC1 channels. On the other hand, Ca^2+^ entry through TRPC1 channels could facilitate PKC, which blocks KCNQ channels. Thus, 2-APB-mediated inhibition of TRPC1 channels may attenuate PKC activation and subsequently also suppress the ghrelin-mediated effect which depends on the PKC-KCNQ channel pathway. For these reasons, treatment of 2-APB or XE991 profoundly suppressed the ghrelin-induced facilitation of MLI firing.

MLIs participate in cerebellar information processing and neural plasticity by regulating the patterns of simple spikes of PCs via GABAergic transmission between MLIs and PCs^18–20^. MLIs form feed-forward inhibition, in addition to a feedback inhibitory circuit, one of which is the Lugaro/globular cell-MLI-PC^48–51^ and keep excitatory/inhibitory balance in the cerebellar cortex^21^, thereby regulates the gain and timing of cerebellar motor learning, including reward-associated learning^20,52–59^. During locomotor behavior, slight alterations in the PC-dendritic excitation/inhibition regulate bidirectionally the firing rate of PCs^59^. Thus, ghrelin-mediated facilitation of GABAergic transmission without changing excitatory synaptic transmission, which we observed here, could cause the modulation of voluntary locomotion dependent on the animal’s nutritional states. In the CNS, ghrelin acts on the hypothalamic system, mesolimbic system connecting the ventral tegmental area (VTA) to the striatum, and hippocampus to control food intake, neurogenesis, and cognitive behavior^60,61^. This peptide enhances the excitability of dopaminergic neurons in the VTA^11^, resulting in the release of dopamine to the nucleus accumbens and facilitating locomotor activity^62^. Elevated dopamine levels could enhance the motivation for feeding behavior and simultaneously improve motor learning, which is valuable for efficient food seeking and taking. Although additional studies are needed to understand ghrelin levels in the cerebellar cortex during fasting, it is possible that elevated ghrelin may control activity in cerebellar circuits by facilitating PC firing, which is accompanied by GABAergic transmission fine-tuned by ghrelin, and subsequently attenuate the excitability of neurons in the deep cerebellar nuclei (DCN). The activity of DCN neurons has recently been reported to control satisfaction and termination of food intake^63^. Thus, the suppression of the membrane excitability of DCN neurons could decrease basal dopamine levels in the ventral striatum and elevate the reward value of food intake^63^. Therefore, sustained fasting may promote appetitive behavior via the effects of ghrelin on cerebellar neuronal activity, possibly accompanied by controlling cerebellar function.

## Materials and methods

### Animals

We used 66 C57BL/6 mice aged 16–28 days of either sex. The mice were housed at our animal facility under a constant temperature and a regular light/dark cycle with free access to food and water.

### Ethical approval for animal experiments

All the experimental procedures were performed in strict accordance with the *Guide for the Care and Use of Laboratory Animals* described by the National Institutes of Health and adhered to the ARRIVE guidelines. These experimental procedures were approved and overseen by the Animal Research Committees on the Care and Use of Animals in Experiments at Wakayama Medical University. In addition, the minimum number of required animals was used for these animal experiments, and efforts were made to minimize pain.

### Electrophysiological recordings

Mouse cerebellar slices were prepared as previously described^64^. The mice were deeply anesthetized via isoflurane inhalation and then decapitated. Sagittal slices (250-μm thick) of the cerebellar vermis were obtained using a vibrating microtome (catalog number VT1200S, Leica) in an ice-cold extracellular solution containing (in mM) 252 sucrose, 3.35 KCl, 21 NaHCO_3_, 0.6 NaH_2_PO_4_, 9.9 glucose, 0.5 CaCl_2_, and 10 MgCl_2_ and gassed with a mixture of 95% O_2_ and 5% CO_2_ (pH 7.4). The slices were maintained at 30 ºC for 30 min in a holding chamber, where they were submerged in ACSF containing (in mM) 138.6 NaCl, 3.35 KCl, 21 NaHCO_3_, 0.6 NaH_2_PO_4_, 9.9 glucose, 2 CaCl_2_, and 1 MgCl_2_ (bubbled with 95% O_2_ and 5% CO_2_ to maintain the pH at 7.4). Thereafter, the slices were maintained at room temperature. Individual slices were transferred to a recording chamber attached to the stage of a microscope (catalog number BX51WI, Olympus) and superfused with oxygenated ACSF. Recordings were performed from PCs and MLIs located exclusively in lobules IV–VII to limit the variability associated with the specialization of different regions of the cerebellar cortex. Spike activity in PCs and MLIs was observed using loose cell-attached voltage-clamp recordings, which allowed long recordings without changing cytoplasmic content^65^. Glass electrodes (2–3 MΩ) used for cell-attached recordings were filled with ACSF and gently placed in contact with PCs and interneurons located in the molecular layer. Slight suction was applied, and the holding potential was set to 0 mV. Here, we did not identify each interneuron as either a basket or a stellate cell according to the criteria for their morphology and physiology^18^, thus, we generically referred to the cells as MLIs. To inhibit synaptic transmission onto MLIs and PCs, we bath-applied the synaptic blockers, 100 μM PTX, 5 μM NBQX, and/or 15 μM APV.

IPSCs of PCs were examined using whole-cell voltage-clamp recordings with patch pipettes (2–3 MΩ). A non-selective ionotropic glutamate receptor antagonist kynurenic acid (1 mM) was added to the ACSF throughout the IPSC recordings. At the first experiment of each data set, we examined whether IPSCs were completely inhibited by bicuculline (10 μM) or PTX (100 μM). To isolate spontaneous IPSCs as outward current responses, patch pipettes were filled with an intracellular solution containing (in mM) 120 K-gluconate, 9 KCl, 10 KOH, 4 NaCl, 10 Na-HEPES, 17.5 sucrose, 10 phosphocreatine, 0.6 QX-314, 3 Mg-ATP, and 0.4 Na-GTP (pH 7.4), and the holding potential was set at − 35 mV. To detect miniature IPSCs (mIPSCs) as larger inward current responses, we used a CsCl-based internal solution (in mM) 140 CsCl, 0.1 CaCl_2_ 1 K-EGTA, 10 Na-HEPES, 10 phosphocreatine, 0.6 QX-314, 3 Mg-ATP, and 0.4 Na-GTP (pH 7.4), and the holding potential was set at − 60 mV in the presence of tetrodotoxin (TTX, 0.5 μM). Stimulation-evoked IPSCs (eIPSCs) were recorded using a cesium methanesulfonate-based internal solution (in mM) 140 CsCH_3_SO_3_, 5 CsCl, 0.1 CaCl_2_ 1 K-EGTA, 10 Na-HEPES, 10 phosphocreatine, 0.6 QX-314, 3 Mg-ATP, and 0.4 Na-GTP (pH 7.4), and the holding potential was set at 10 mV. Focal stimulation (20–50 V, 0.1 ms) was applied using a glass microelectrode containing ACSF (1–2 MΩ) placed within the ML of the cerebellar slices. PF-EPSCs were recorded using the cesium methanesulfonate-based internal solution and the holding potential was − 60 mV. Focal stimulation (20–50 V, 0.05–0.1 ms) was applied via a glass microelectrode containing ACSF (1–2 MΩ) placed within the ML of the cerebellar slices. Paired-pulse stimulation was delivered at an interval of 7.5 s in the presence of PTX (100 μM). We did not correct the junction potential.

Series and input resistances were monitored continuously online with 2-mV hyperpolarizing voltage steps at an interval of 7.5 or 30 s. Series resistance (10–18 MΩ) was compensated by 60–70%, and the experiments were discarded if the value changed by ~ 20%. Experiments were performed at room temperature (24–26 °C). TTX was obtained from FUJIFILM Wako Pure Chemical Industries (Osaka, Japan), JMV3002 (Cayman Chemical, Ann Arbor, MI), 2-APB (Abcam Biochemicals, Cambridge, UK), CGP55845, QX-314 and XE991 (Tocris Bioscience, Bristol, UK), and all other chemicals were purchased from Sigma-Aldrich (St. Louis, MO). The membrane currents were recorded using an amplifier MultiClamp 700B (Molecular Devices, Sunnyvale, CA) and pCLAMP 10.3 software (Molecular Devices), digitized, and stored on a computer disk for offline analysis. All signals were filtered at 2–4 kHz and sampled at 5–20 kHz, and synaptic events were analyzed with a threshold of 10 pA. The frequencies of synaptic events are shown as the number of synaptic events (for 30 s) divided by the time duration. Spike firing and synaptic events were analyzed using the Mini Analysis Program 6.0 (Synaptosoft, Decatur, GA), Clampfit 10.3 software (Molecular Devices), and KyPlot software (version 6.0; KyensLab, Tokyo, Japan).

### Experimental design and statistical analysis

Throughout the manuscript, all data were expressed as mean ± SEM. All statistical analyses were performed using the KyPlot software (version 6.0; KyensLab). Throughout the data analyses of electrophysiological signals, *n* refers to the number of recorded neurons, and the number of slices and animals are shown as slices/mice (i.e., *n* = 8 from 7/6 indicates that eight neurons were recorded from seven slices of six mice). All electrical signals were analyzed using Mini Analysis Program 6.0 (Synaptosoft; RRID:SCR_002184), Clampfit 10.3 software (Molecular Devices), and KyPlot software (version 6.0; KyensLab). Unless otherwise stated, the level of significance was determined using the Wilcoxon signed-rank test or the Mann–Whitney U test for comparisons between related and independent sample groups. Power analysis was performed using G*Power (v3.1.9.6, www.gpower.hhu.de). A prior power analysis was used to obtain the minimum number of sample sizes and animals for adequate study power to detect differences among groups.

## Abbreviations

ACSF: Artificial cerebrospinal fluid
eIPSC: Evoked inhibitory postsynaptic current
IPSC: Inhibitory postsynaptic current
CNS: Central nervous system
CV: Coefficient of variation
CV2: Coefficient of variation 2
GABA: γ-Aminobutyric acid
GHS-R1a: Growth hormone secretagogue receptor 1a
GIRK: G-protein-coupled inward-rectifying K^+^
mIPSC: Miniature inhibitory postsynaptic current
MLI: Molecular layer interneuron
PPR: Paired-pulse ratio
PC: Purkinje cell
sIPSC: Spontaneous inhibitory postsynaptic current
TRPC1: Type 1 transient receptor potential canonical
